# Smoking Cessation Outcomes in an Inner-City Interdisciplinary Pulmonary Clinic Involving Ambulatory Care Pharmacists and Pulmonary Specialists: A Retrospective Observational Study

**DOI:** 10.7759/cureus.117781

**Published:** 2026-10-11

**Authors:** Wyanna Yeung, Kristen Mathew, Gilda Diaz-Fuentes, Charnicia Huggins

**Affiliations:** 1 Pharmacy, BronxCare Health System, Bronx, USA; 2 Pulmonary and Critical Care, BronxCare Health System, Bronx, USA

**Keywords:** ambulatory care pharmacist, pharmacist-led intervention, pulmonary specialist, smoking abstinence, smoking cessation

## Abstract

Introduction: Tobacco smoking remains one of the leading causes of preventable pulmonary disease and contributes significantly to morbidity and mortality. Although smoking cessation has been shown to improve lung function, reduce hospitalization, and decrease mortality risk, smoking cessation rates continue to be suboptimal. These findings highlight the ongoing need for increased access to and utilization of effective smoking cessation interventions to improve long-term cessation outcomes. The primary outcome of the study was to describe smoking cessation rates among patients receiving pharmacist-led interventions. Secondary outcomes included smoking cessation rates comparing individuals who used pharmacotherapy versus those who did not, as well as changes in cigarettes smoked per day and adherence to pharmacotherapy among patients who did not achieve abstinence.

Methods: This was a retrospective study of patients who received smoking cessation services as part of a performance improvement project. At the adult pulmonary clinic, patients who smoked and were willing to quit were referred by the pulmonologist to the clinical pharmacists for management and counseling. The referral period was August to December 2025, and patients were followed for an additional two months. Pharmacists conducted comprehensive smoking cessation counseling and assessment via the Fagerström Test for Nicotine Dependence, selected appropriate pharmacotherapy, or recommended alternative therapy as needed. Follow-up phone calls were also conducted to assess medication adherence, monitor for adverse effects, and provide ongoing support and encouragement throughout the smoking cessation process. A chi-square test was used to test statistical significance, with a p-value threshold of <0.05.

Results: A total of 40 patients, predominantly Hispanic or African American, were referred; 25 (62.5%) received either behavioral counseling alone or a combination of behavioral counseling and pharmacotherapy. Four (16%) of those patients achieved smoking abstinence, six (24%) patients exhibited an improvement in their smoking behavior, while 14 (56%) patients had no change in smoking status. One (4%) patient experienced worsening tobacco use during the study period.

Conclusions: A multidisciplinary approach to smoking cessation involving pulmonologists and clinical pharmacists was associated with positive smoking cessation outcomes. More than one-third of participants receiving behavioral counseling and pharmacotherapy achieved smoking cessation or reduced cigarette consumption; however, direct comparison with published cessation rates is limited by differences in study populations, intervention protocols, follow-up duration, and outcome definitions. These findings suggest that multidisciplinary smoking cessation interventions may be feasible in an inner-city clinic without additional resources.

## Introduction

Tobacco smoking remains one of the leading causes of preventable pulmonary disease and contributes significantly to morbidity and mortality worldwide. Smoking cessation has been shown to improve lung function, reduce hospitalization, and decrease mortality risk. Despite these well-established benefits, smoking cessation rates remain low [1]. According to the U.S. Centers for Disease Control and Prevention (CDC), the smoking cessation rate in 2022 was only 8.8% [2]. Additionally, individuals attempting to quit without a structured approach achieve success rates of only 3% to 5% [3]. Evidence-based smoking cessation treatments continue to be underutilized, with fewer than 40% of adults who smoke using pharmacologic or behavioral treatment strategies during quit attempts [4]. Pharmacotherapy smoking cessation options include varenicline, bupropion, and different forms of nicotine replacement therapy, available as patch, gum, lozenge, and nasal spray [5-7]. Pharmacists can play an important role in the effective delivery of smoking cessation counseling and maintenance of quit attempts through various interventions [8]. A previous study showed that patients with significant pulmonary disease had higher rates of smoking cessation at 12 months when managed by a pharmacist compared with routine counseling provided during practitioner-led visits [9]. These findings highlight the ongoing need for increased access to and utilization of pharmacists in smoking cessation interventions. Additional research is needed due to limited real-world evidence and a need for a diverse population to confirm generalizability. Second, high-risk populations, particularly low-income groups, remain understudied. These patients often face greater barriers to smoking cessation.

## Materials and methods

Design

This was a single-center, retrospective, observational cohort study. Patient data were collected from August 1, 2025, to December 31, 2025, via an electronic medical record; patients were followed for an additional two months afterward. The study was approved by the Institutional Review Board (IRB# 02 12 26 01). Categorical variables were summarized using frequencies and percentages, and the chi-square test was used for categorical comparisons where appropriate. Given the small sample size and limited cell counts, statistical findings were interpreted cautiously. A p-value <0.05 was considered statistically significant [10].

Setting

BronxCare Health System, located in the Bronx, New York, consists of two main hospital divisions and more than 30 outpatient clinics. The Outpatient Center for Pulmonary Medicine provides comprehensive care for a variety of pulmonary diseases, including chronic and complex respiratory conditions such as asthma and chronic obstructive pulmonary disease (COPD). The center offers specialized evaluation, diagnosis, and treatment services aimed at improving respiratory health and patient outcomes through evidence-based, multidisciplinary care.

Objective

The primary outcome of the study was to describe smoking cessation rates, defined as self-reported abstinence at the final follow-up, among participants referred by pulmonary specialists who received pharmacist-led interventions. Secondary outcomes included smoking cessation rates comparing individuals who used pharmacotherapy versus those who did not, as well as changes in cigarettes smoked per day and adherence to pharmacotherapy among participants who did not achieve abstinence. Improvement in smoking behavior was defined as a reduction in cigarette consumption without complete abstinence, while worsening was defined as an increase in cigarette consumption compared with baseline. These secondary analyses aimed to further explore the impact of pharmacologic support on cessation success and to observe smoking behavior and treatment response in individuals who were unable to quit.

Study participants

Study eligibility criteria include adults at least 18 years of age who were current smokers (defined as any cigarette use within the past 30 days) and established patients of the pulmonary clinic from August 1, 2025, to December 31, 2025. Patients who were unwilling to receive smoking cessation counseling were excluded.

Intervention

Patients were referred to the clinical pharmacist for smoking cessation counseling after the pulmonologist provided an initial discussion regarding smoking cessation to the patient and identified those who were willing to quit smoking. During the initial visit, pharmacists provided comprehensive smoking cessation counseling using the 5A’s framework (Ask, Advise, Assess, Assist, and Arrange) for patients ready to quit and the 5R’s framework (Relevance, Risks, Rewards, Roadblocks, and Repetition) for those not yet ready to quit. Nicotine dependence was also evaluated using the Fagerström Test for Nicotine Dependence. Based on the patient’s smoking history, readiness to quit, and level of nicotine dependence, appropriate smoking cessation therapy was selected. Pharmacotherapy treatment included varenicline, bupropion, and nicotine replacement therapy (i.e., patch, gum, lozenge, or nasal spray). Pharmacists communicated treatment recommendations to the pulmonary specialists, and prescriptions were sent to the patient’s pharmacy. Follow-up phone calls were conducted to assess medication adherence, monitor for adverse effects, and provide ongoing support and encouragement throughout the smoking cessation process. Patients not contacted after three telephone attempts were considered lost to follow-up. Patients successfully contacted were followed up on a monthly basis from the initial clinic visit until February 28, 2026, as an aspect of performance improvement.

## Results

A total of 40 patients were referred for smoking cessation; 6 patients declined participation, and 34 patients received pharmacist-led counseling. Among those counseled, nine patients declined pharmacotherapy (Figure 1).

**Figure 1:**
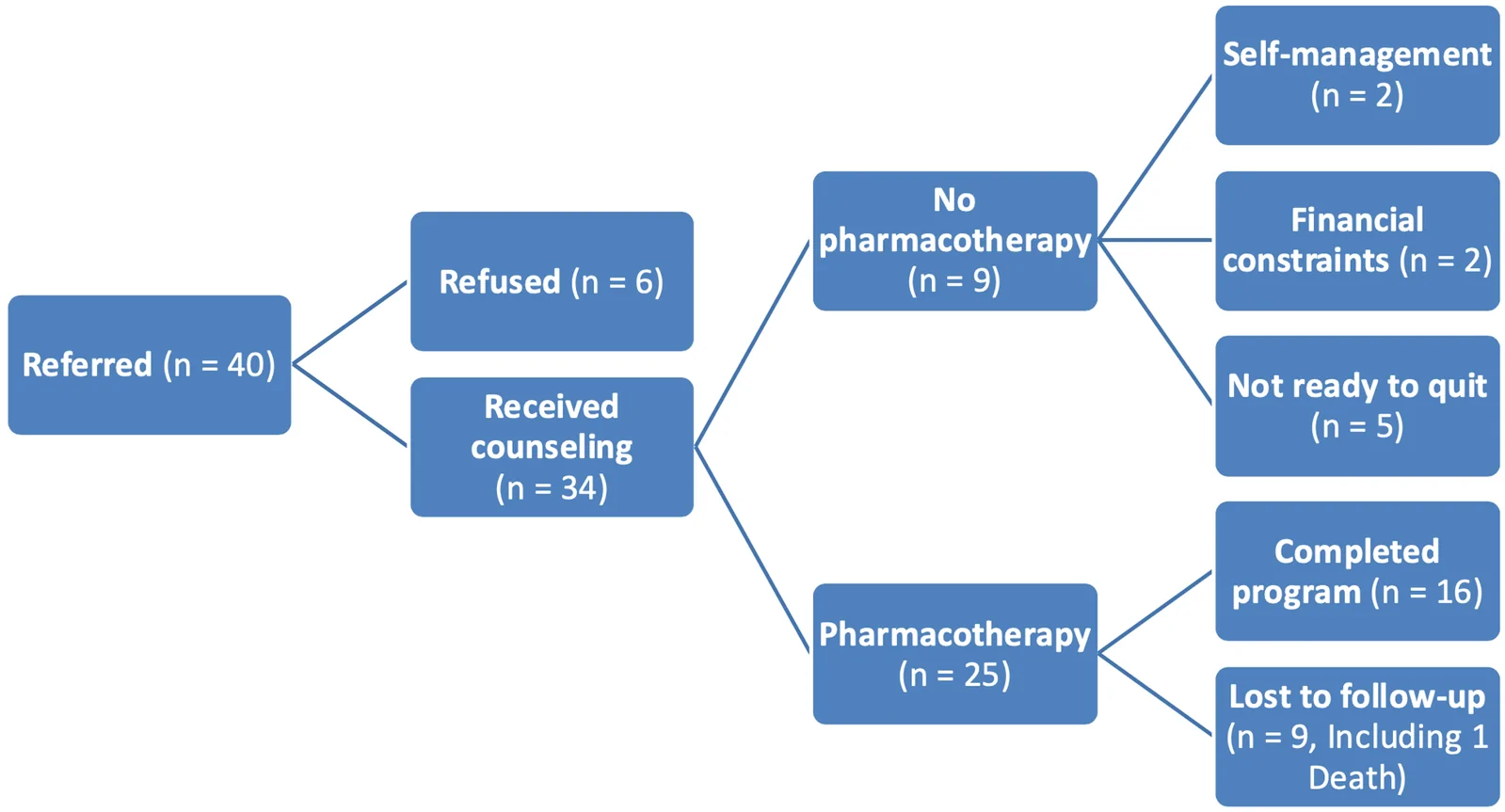
Flowchart of the study population screening, inclusion, and exclusion process

The primary reasons included preference for self-management, financial constraints, and unwillingness to quit. Most patients in our cohort were female (65%), with a median age of 64 years, and 50% were Hispanic or Latino. The most important comorbidity was the presence of COPD in 82% of patients and psychiatric disorders in 35% (Table 1).

**Table 1:** Demographic and clinical characteristics of the study population COPD, chronic obstructive pulmonary disease.

	Pharmacotherapy (n=25)	No pharmacotherapy (n=9)	p-value​
Sex, n (%)​			
Female	13 (52)	9 (100)	0.0098​
Male	12 (48)​	0 (0)​	
Age in years, median (range)	66 (42-87)​	62 (47-69)​	-​
Race, n (%)​			
Black or African American	9 (36)​	5 (55.6)​	0.3067​
White or Caucasian	0​ (0)	0​ (0)	-
Other	15 (60)​	4 (44.4)​	0.6496​
Unknown	1 (4)​	0​ (0)	0.7384​
Ethnicity, n (%)​			
Hispanic or Latino​	13 (52)​	4 (44.4)​	0.6974
Not Hispanic or Latino	11 (44)​	5 (55.6)	0.5515​
Unknown​	1 (4)​	0​ (0)	0.5425​
Comorbidities, n (%)​			
COPD and/or asthma​	21 (84)​	7 (77.8)​	0.6746​
Psychiatric disorders​	8 (32)​	4 (44.4)​	0.5029​
Insurance, n (%)​			
Medicare/Medicaid​	24 (96)​	8 (88.9)​	0.4369​
Commercial​	1 (4)​	1 (11.1)​	0.4369​

In terms of the primary outcome, 25 (62.5%) patients initiated pharmacotherapy and received pharmacist-provided counseling (Table 2); 16 (64%) of 25 were successfully contacted via follow-up by the pharmacist, and 9 (36%) patients were lost to follow-up, including one reported death. Among the 25 patients who received pharmacist counseling, with or without pharmacotherapy initiation, and completed follow-up calls, 4 (16%) achieved smoking abstinence. Improvement in smoking behavior was observed in 6 of 25 (24%) patients, while 14 (56%) had no change in smoking status. Only 1 (4%) patient experienced worsening tobacco use during the program (Table 3).

**Table 2:** Pharmacotherapy selection NRT, nicotine replacement therapy.

Pharmacotherapy	Patients (n=25)
NRT alone, n (%)	8 (32)
Varenicline alone, n (%)	1 (4)
Bupropion alone, n (%)	0 (0)
Combination pharmacotherapies, n (%)	16 (64)
Varenicline + nicotine patch	4 (25)
Varenicline + nicotine gum	0 (0)
Varenicline + nicotine lozenge	1 (6.2)
Nicotine patch + nicotine gum	7 (43.8)
Nicotine patch + nicotine lozenge	4 (25)

**Table 3:** Smoking cessation outcomes between intervention groups

	Abstinence​, n (%)	Improvement, n (%)​	Worsening, n (%)​	Unchanged​, n (%)	p-value​
Among participants receiving pharmacist-led interventions					
Received counseling and completed program (n=25)	4 (16)​	6 (24)​	1 (4)	14 (56)​	-
Stratified by pharmacotherapy use					
Pharmacotherapy​ (n=16)	4 (25)​	5 (31.3)​	0​	7 (43.7)​	0.1026​
No pharmacotherapy​ (n=9)	0​	1 (11.1)​	1 (11.1)​	7 (77.8)	

Although complete cessation was modest, a meaningful proportion of patients demonstrated some level of benefit from the intervention. Overall, abstinence or improvement in smoking behavior was observed among participants in the pharmacist-led smoking cessation program. The secondary outcome also evaluates smoking cessation rates stratified by pharmacotherapy use. No patients in the pharmacotherapy group experienced an increase in cigarette use, whereas one patient in the non-pharmacotherapy group reported an increase in daily cigarette consumption (Table 3). Nonetheless, as an exploratory observation, among these patients who did not achieve abstinence, three in the pharmacotherapy group experienced a 50% or greater reduction in cigarette use, whereas none in the non-pharmacotherapy group achieved a similar decrease in cigarettes smoked per day (Table 4). However, given the small sample size and lack of randomization, these findings should be interpreted cautiously.

**Table 4:** Cigarette reduction and medication adherence in non-abstinent participants

​	Pharmacotherapy​ (n=12)​, n (%)	No pharmacotherapy​ (n=9)​, n (%)
≥50% decrease in cigarettes smoked per day	3 (25)​	0 (0)​
Adherence to pharmacotherapies	4 (33)​	-​

## Discussion

The abstinence rate observed in our study (16%) was comparable to rates reported in previous smoking cessation programs. One pharmacist-led study, Andras et al., reported a 12.8% quit rate at six months among patients managed by pharmacists, compared with 11.4% in the practitioner-managed cohort [9]. Similarly, another pharmacist-led tobacco cessation program, Shen et al., reported an average six-month point prevalence quit rate of 18.0% [11]. A physician-led smoking cessation program, Byers et al., also demonstrated a comparable abstinence rate of 18.2% [12]. Collectively, these findings are consistent with previously reported benefits of structured smoking cessation interventions across healthcare settings and disciplines; however, the present study did not demonstrate a statistically significant treatment effect. Our findings support the feasibility of pharmacist involvement in tobacco cessation through behavioral counseling, pharmacotherapy management, and longitudinal follow-up; however, larger controlled studies are needed to determine its effectiveness and further evaluate the role of pharmacists in improving tobacco cessation outcomes.

Smoking cessation has been consistently associated with improved pulmonary outcomes, including increased lung function, reduced respiratory symptoms, fewer hospitalizations, and decreased mortality risk. These benefits are especially important for patients with chronic pulmonary diseases, in whom continued tobacco use can accelerate disease progression and worsen quality of life [13,14]. Pulmonary clinics represent critical opportunities for smoking cessation interventions because patients may often be more aware of the impact of smoking on their respiratory health, due to potential diagnoses of smoking-related illnesses, and may be more motivated to quit. However, despite the importance of tobacco cessation counseling, physician time constraints and competing clinical priorities can limit the ability to provide comprehensive smoking cessation support during routine visits.

Pharmacists are well positioned to help address this gap in care through collaborative, multidisciplinary smoking cessation services. Pharmacist-led interventions include behavioral counseling, assessment of readiness to quit, evaluation of nicotine dependence, and individualized selection and management of pharmacotherapy such as nicotine replacement therapy, bupropion, or varenicline. In addition, pharmacists can provide medication education, monitor adherence and adverse effects, and offer regular follow-up support to reinforce quit attempts and prevent relapse. Incorporating pharmacists into pulmonary clinic practices may improve access to evidence-based treatment and enhance patient outcomes through more comprehensive and continuous support.

The study included a predominantly minority patient population from a single inner-city pulmonary clinic, providing real-world data from a group that is often underrepresented in clinical research; however, our single-center design and small sample limit generalizability. A key strength of our study was the involvement of an interdisciplinary team of pharmacists and pulmonary specialists, reflecting a collaborative care model. Nevertheless, our reliance on self-reported smoking cessation outcomes coupled with the high attrition rate of our study and the lack of a control group may impact the reliability of the findings. In addition, the absence of statistically significant findings highlights the need for larger, more robust future studies.​

## Conclusions

Pharmacist involvement was associated with favorable smoking cessation outcomes, with pharmacists providing access to evidence-based interventions and consistent patient support throughout the quitting process. Through individualized counseling, assessment of nicotine dependence, pharmacotherapy management, and follow-up monitoring, pharmacists can play an important role in helping patients successfully reduce or quit tobacco use. Participants who received either behavioral counseling alone or a combination of behavioral counseling and pharmacotherapy had observed abstinence or improvement in smoking behavior in this study. These findings highlight the value of incorporating pharmacists into multidisciplinary pulmonary and outpatient care settings to enhance smoking cessation services, optimize treatment utilization, and improve long-term respiratory and overall health outcomes. Finally, more robust evidence is necessary to support broader implementation of smoking cessation services and to influence healthcare policy and funding.
